# Measles outbreak propagated by children congregating at water collection points in Mayuge District, eastern Uganda, July – October, 2016

**DOI:** 10.1186/s12879-018-3304-5

**Published:** 2018-08-20

**Authors:** Robert Kaos Majwala, Lydia Nakiire, Daniel Kadobera, Alex Riolexus Ario, Joy Kusiima, Joselyn Annet Atuhairwe, Joseph K. B. Matovu, Bao-Ping Zhu

**Affiliations:** 1 Uganda Public Health Fellowship Program - Field Epidemiology Track, Ministry of Health of Uganda and Makerere University School of Public Health, P.O. Box 7072, Kampala, Uganda; 2 National Tuberculosis and Leprosy Program, Ministry of Health Uganda, Kampala, Uganda; 3 Makerere University School of Public Health, Kampala, Uganda; 4 Centers for Disease Control and Prevention, Atlanta, GA USA

**Keywords:** Disease outbreaks, Measles, Risk factors, Uganda

## Abstract

**Background:**

On 12 October, 2016 a measles outbreak was reported in Mayuge District, eastern Uganda. We investigated the outbreak to determine its scope, identify risk factors for transmission, evaluate vaccination coverage and vaccine effectiveness, and recommend evidence-based control measures.

**Methods:**

We defined a probable case as onset of fever (≥3 days) and generalized rash, plus ≥1 of the following: conjunctivitis, cough, and/or runny nose in a Mayuge District resident. A confirmed case was a probable case with measles-specific IgM (+) not explained by vaccination. We reviewed medical records and conducted active community case-finding. In a case-control investigation involving probable case-persons and controls matched by age and village, we evaluated risk factors for transmission for both cases and controls during the case-person’s likely exposure period (i.e., 7–21 days prior to rash onset). We estimated vaccine effectiveness (VE) using the formula: VE ≈ (1-OR_protective_) × 100. We calculated vaccination coverage using the percentage of controls vaccinated.

**Results:**

We identified 62 probable case-persons (attack rate [AR] = 4.0/10,000), including 3 confirmed. Of all age groups, children < 5 years were the most affected (AR = 14/10,000). The epidemic curve showed a propagated outbreak. Thirty-two percent (13/41) of case-persons and 13% (21/161) of control-persons visited water-collection sites (by themselves or with parents) during the case-persons’ likely exposure period (OR_M-H_ = 5.0; 95% CI = 1.5–17). Among children aged 9–59 months, the effectiveness of the single-dose measles vaccine was 75% (95% CI = 25–92); vaccination coverage was 68% (95% CI = 61–76).

**Conclusions:**

Low vaccine effectiveness, inadequate vaccination coverage and congregation at water collection points facilitated measles transmission in this outbreak. We recommended increasing measles vaccination coverage and restriction of children with signs and symptoms of measles from accessing public gatherings.

**Electronic supplementary material:**

The online version of this article (10.1186/s12879-018-3304-5) contains supplementary material, which is available to authorized users.

## Background

Measles is a highly contagious infectious disease [1] that spreads efficiently from person to person via the respiratory route [2], including airborne transmission [3]. It is one of the top five causes of vaccine preventable morbidity and mortality in the world [4]. The incubation period from exposure to fever varies from 7 to 21 days and from exposure to rash usually about 14 days [1, 5–7]. Due to the availability of a highly effective and low-cost vaccine and the fact that the disease does not have non-human reservoirs, measles has been targeted for elimination and eventual eradication [8]. Rapid detection of public health threats such as outbreaks of measles, and control of such threats at their source, are essential for global health security.

Uganda began to implement routine measles vaccination in the early 1980’s through static clinics at health facilities and outreach posts in the community. Supplemental measles mass vaccination has also been provided routinely every 3 years since 2003. Measles surveillance in Uganda is part of the National Integrated Disease Surveillance and Response System, which requires immediate notification whenever a suspected measles case is identified [8, 9]. When a measles case is suspected, a case investigation form is completed and blood samples are collected and submitted to the Uganda Virus Research Institute (UVRI) for testing [8].

In October 2016, a suspected measles outbreak occurred in Mayuge District, eastern Uganda. Of the 10 blood samples collected from suspected case-persons, three tested positive for measles IgM antibodies at UVRI. The Uganda Ministry of Health (MoH) declared a measles outbreak in the District.

**Fig. 1:**
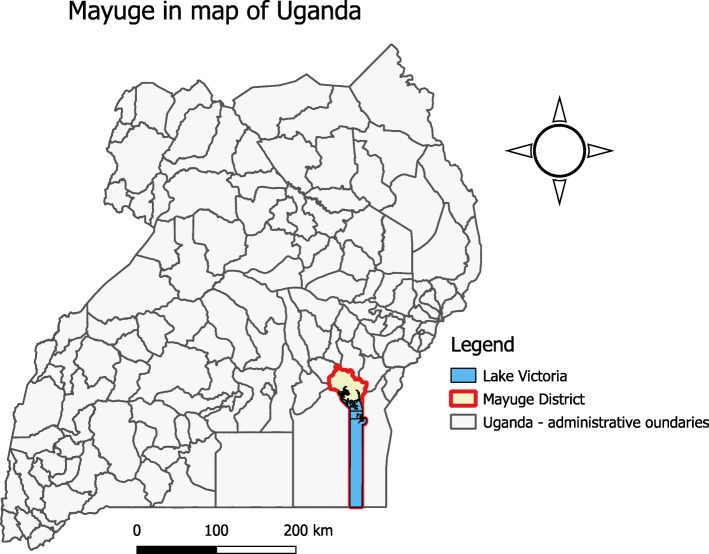
Location of Mayuge District in Uganda where a measles outbreak occurred: October 2016, the figure was constructed using Quantum Geographic Information System version 2.8.1 (QGIS 2.8.1)

Mayuge District (0°27′28.0″N, 33°28′48.0″E) is located in Eastern Uganda. The district is located about 120kms from Kampala, Uganda’s capital. It is bordered by Iganga District to the North, Jinja in the West, Bugiri in the East and Lake Victoria in the South. The District has 13 sub-counties (Fig. 1), and had a total population of 480,079, of which 4.3% (20,643) were children less than 1 year of age [10].

We conducted an investigation to determine the extent of the outbreak, identify risk factors, estimate vaccine effectiveness and vaccination coverage, and recommend evidence-based control measures.

## Methods

We defined a probable case as onset in a resident of Mayuge District of fever and generalized rash with at least one of the following symptoms: coryza, conjunctivitis, or cough, between 19 June and 17 November, 2016. A confirmed case was a probable case with measles IgM (+) in the absence of vaccination in the preceding 2 weeks.

We actively searched for case-persons in the community with the help of community leaders, village health team members and health assistants (i.e., a cadre of public health workers at parish level involved in disease surveillance [11]). We reviewed health facility records at community health centers in the outbreak area. Using a standardized case investigation form, we collected data on case-person’s demographics, clinical information, and exposure history.

We assessed the time distribution of measles cases by constructing epidemic curves. We computed measles attack rates by person and place characteristics. In calculating the attack rates, we estimated the Mayuge District population by extrapolating from the 2014 Uganda National Population Census using the district-specific population growth rates [10].

Laboratory confirmation was conducted at the Expanded Program for Immunization Laboratory (EPI Lab) at UVRI, using the recommended World Health Organization (WHO) procedures [12].

### Case-control investigation

We conducted hypothesis-generating interviews of 20 measles case-persons in the parish with the highest attack rate. We asked the case-persons about potential risk factors for measles transmission between 7 and 21 days prior to symptom onset, including visits to health facilities, community playgrounds, and water-collection points; attendance at place of worship, schools, burial events, and ‘village saving group meetings’ (locally referred to as *Nigina*); and travel history.

To test the hypotheses developed during the hypothesis-generating interviews, we conducted a case-control investigation (Additional file 1) in the two affected sub-counties. The case-control study was conducted in children aged 6–59 months because most of the cases were in this age group. We administered the questionnaire to caregivers or guardians since the case-persons were all minors. We selected one case-person per household; for households with more than one case-person, the first to develop a rash was selected for interview. For each case-person, 4 asymptomatic controls were randomly selected among residents of the same village, matched by age (±12 months).

### Vaccination coverage and vaccine effectiveness

We estimated vaccination coverage (VC) using the percent of control-persons vaccinated. Vaccination status was obtained from immunization records obtained from care takers (immunization cards). We also obtained the administrative data on the number of doses of measles vaccines administered reported in the District Health Information Software, version 2 (DHIS2) for Mayuge District during 2016. In estimating the administrative VC, we used the population estimates for Mayuge District provided by the Uganda National Population Census report 2016 [10].

We estimated Vaccine effectiveness (VE) using the following formula:

$$ VE=1-{RR}_{\mathit{\Pr} otective}\approx 1-{OR}_{\mathit{\Pr} otective} $$

where *RR*_*Protective*_ is the protective relative risk associated with vaccination, which can be approximated by the protective odds ratio,*OR*_*Protective*_, in rare diseases such as measles.

We estimated VC and VE in children aged 9–59 months because Uganda’s routine measles vaccination starts at 9 months of age and this outbreak mainly affected children aged ≤59 months.

## Results

### Descriptive epidemiology

We identified 62 case-persons (overall attack rate [AR] =1.3/10,000), including 3 confirmed by measles-specific IgM. The median age of the case-persons was 35 months (range: 4 months to 50 years). The most affected age groups were 0–11 months (AR = 13/10,000) and 12–59 months (AR = 15/10,000). The attack rate was similar between male (4.3/10,000) and female residents (AR = 3.9/10,000). The outbreak affected 2 sub-counties, Kityerera (AR = 4.1/10,000) and Malongo (AR = 4.0/10,000). The most affected parish was Bumwena (AR = 79/10,000) followed by Kityerera (AR = 13/100,000) (Table 1). None of the case-persons exhibited any complications.

**Table 1:** Measles attack rate by age, sub-county, sex, and parish during a measles outbreak: Mayuge District, Uganda, July – October 2016

Characteristics/Variable		Population	Cases	AR/10,000
Age (Months)	< 11	6672	9	13
	12–59	24,671	38	15
	> 60+	123,818	15	1.2
Sub county	Kityerera	49,330	20	4.1
	Malongo	105,831	42	4.0
Sex	Males	73,736	32	4.3
	Females	76,810	30	3.9
Parishes in Malongo Sub-county	Bumwena	2649	21	79
	Bukatabira	5167	4	7.7
	Malongo	17,971	10	5.6
	Namoni	5790	3	5.2
	Buluuta	11,852	3	2.5
	Namadhi	6373	1	1.6
	Bukalenzi	11,500	1	0.87
Parishes in Kityerera Sub-county	Kityerera	13,449	19	14
	Maumu	4392	1	2.3

**Fig. 2:**
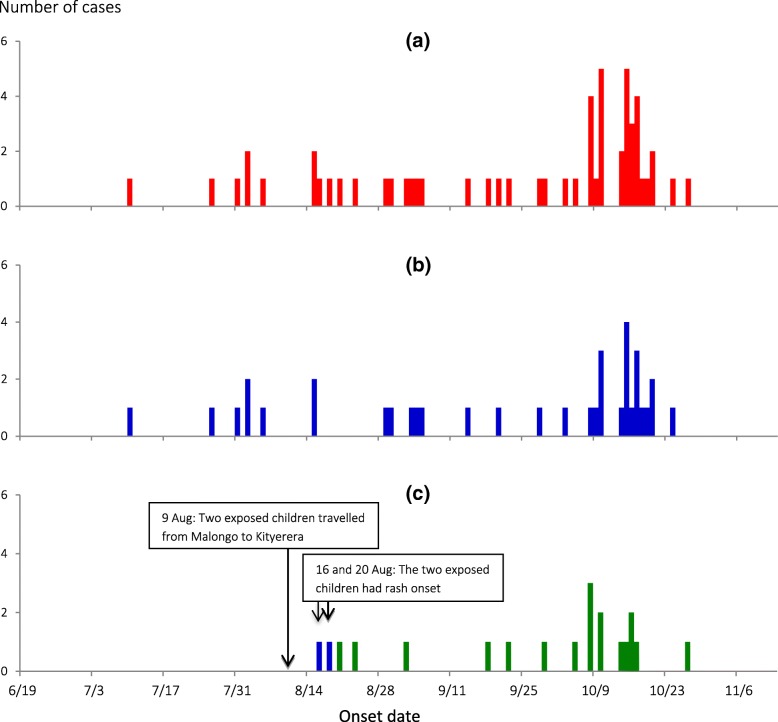
Date of rash onset among measles case persons June – November, 2016. **a** shows district epidemic curve, **b** epidemic curve for Malongo Sub-county and **c** epidemic curve for Kityerera Sub-county

The epidemic curve indicated person-to-person transmission (Fig. 2a ). From the symptom onset date of the index case-person (10 July) to that of the last case-person (27 October), the outbreak lasted 110 days. When the epidemic curve was stratified by sub-county, both Malongo Sub-county (Fig. 2b ) and Kityerera Sub-county (Fig. 2c) show propagated outbreaks. The outbreak first started in Malongo Sub-county in July, with several subsequent generation periods. On 9 August, 2 children, aged 24 and 36 months, from a family with suspected measles cases travelled with their mother from Bumwena Parish, Malongo Sub-county to Kityerera Parish, Kityerera Sub-county. On 16 August, 7 days after they arrived in Kityerera Parish, the 36-month-old toddler developed a rash; on 20 August, the younger sibling also developed a rash. Subsequently, a case occurred in the new sub-country, ultimately affecting 18 children. The hypothesis-generating interviews pointed to attendance of social and religious gatherings, school attendance and going to water-collection points as possible risk factors.

### Case-control investigation findings

Among children aged 6–59 months, 32% (10/31) of the case-persons, compared with 13% (16/122) of control-persons, went to water-collection points either alone, with their parents/guardians or with other children (OR_MH_ = 5.0, 95% CI: 1.5–17) (Table 2). No other risk factors examined in the case-control investigation was significantly associated with measles onset.

**Table 2:** Risk factors for measles infection among children aged 6–59 months during a measles outbreak Mayuge District, Uganda, July – October 2016

Risk factor	% of cases exposed (*n* = 31)	% of controls exposed (*n* = 121)	OR_MH_ (95% CI)
Went to water-collection points	32	13	5.0 (1.5–17)
Visited church	48	32	2.1 (0.91–4.9)
Visited health facility	13	18	0.62 (0.20–1.9)
Attended mass vaccination	16	28	0.45 (0.14–1.5)

Village health team members reported that in this community mothers with infants < 6 months of age do not go to usually collect water. Mothers who have infants aged 6 months – 2 years tend to carry their babies along as they go to collect water. Children aged > 2 years often go together with their mothers, guardians or other siblings to water-collection points, often carrying age-appropriate water-collection vessels.

### Measles vaccine effectiveness

When the case-control investigation data were analyzed for children aged 9–59 months, 39% (12/31) of case-persons compared to 68% (82/121) of controls had a history of measles vaccination (OR_MH_ = 0.25; 95% CI: 0.12–0.75). Using this information, we estimated that VE = 75% (95% CI: 25–88%) in children aged 9–59 months.

### Vaccination coverage

Of controls aged 9–59 months, 68% (95% CI: 61–76%) had a history of vaccination; this percentage was the estimated VC. Based on the administrative data, the cumulative (January–September, 2016) measles VC in Mayuge District in September 2016 was 52% against the expected 75% by extrapolation; if no catch-up vaccination had been implemented, VC would have been 69% by December 2016.

## Discussion

Our investigation showed that this community measles outbreak was propagated by children congregating at water-collection points. The outbreak lasted 110 days with 62 case-persons. Our investigation demonstrated that the outbreak was transmitted from the Bumwena Parish, Malongo Sub-county to the Kityerera Parish, Kityerera Sub-county by 2 exposed children during their incubation period. Congregation of children at water-collection points facilitated measles transmission within the community, whereas low VC and suboptimal VE increased the susceptibility of the population.

Two children who travelled with their mother from Bumwena Parish to Kityerera Parish were the likely source of infection for the outbreak in Kityerera Parish. They had exposure to measles case-persons in the household where they were staying in Bumwena Parish (before they travelled). Since the measles incubation period ranges from 7 to 21 days [6], the 36-month-old child, who developed a rash 7 days after leaving the family with measles cases in Bumwena Parish, likely had exposure in Bumwena Parish, whereas the 24-month-old child, who developed rash 11 days after leaving Bumwena Parish, likely have contracted measles from his older sibling after arriving in Kityerera Parish.

During this outbreak, congregation of children at water-collection points was a strong risk factor for measles infection. In this community, women are the primary caretakers for children; women and children tended to move together to collect water. When they arrive at the water-collection points, the children often spend a lot of time mingling and playing with each other, facilitating the transmission of measles. Congregate settings have been demonstrated to facilitate measles transmission in various settings [13, 14], and hence this explains the importance of discouraging measles susceptible individuals from attending congregate settings that may expose them to measles infection and disease during measles outbreaks.

Just like many other Sub-Saharan African countries, Uganda currently administers a one-dose measles containing vaccine given at 9-months as part of the national vaccination schedule [15]. To achieve maximum protection against measles, studies have recommended measles vaccination at 12 months as opposed to 9 months [16]. The measles coverage in Mayuge was estimated at 69%, is much lower than the recommended vaccination coverage of > 90% required for herd immunity and for achieving the measles elimination goal set by WHO for the African Region and adopted by the Uganda MoH to achieve population immunity by WHO for the African Region [17]. Even in countries where good vaccination coverage has been achieved; measles outbreaks still occur as a result of susceptible individuals accumulating over time.

The estimated VE from our investigation (75%) was lower than the observed 85–94% in other studies for one-dose measles vaccination [18]. Several factors affect VE, including age at vaccination, and vaccine handling techniques, etc. [19]. In a previous study, the VE for a single-dose measles vaccine administered at 12 months of age was 92%, compared with 85% when the vaccine was given at 9 months [18]. A 2-dose measles vaccine with one given at 9 and the other at about 12 months increased VE to over 94% [18]. During the past 2 years, several measles outbreaks have occurred in Uganda, and the VE has been consistently low (Uganda Public Health Fellowship Program, unpublished data). The reason for the low VE in Uganda is not entire clear, but it can be partially explained by the fact that Uganda’s one-dose vaccination is given at 9 months of age.

### Study limitations

In the case control study, we used asymptomatic controls, we did not test the controls for measles IgM antibodies, and hence this might have led to misclassification bias for controls. However control persons were carefully selected based on the case definition above. In this study, we used proportion of controls vaccinated to estimate vaccination coverage instead of standard WHO community survey method. It is possible that with this method, we may not have obtained the exact estimate however we triangulated with the administrative coverage, which yielded the same estimate of 68 and 69%.

## Conclusions

We conclude that exposures to infectious patients at water-collection points propagated this outbreak in Mayuge District. Low VC and poor VE facilitated community transmission of measles in this outbreak.

### Recommendations

Ministries of Health should consider intensification of vaccination coverage among children less than 5 years for achievement of herd immunity to avoid occurrence of measles outbreaks. Mothers and guardians should be discouraged from taking children with measles like symptoms to public gathering.

### Public health actions taken

At our recommendations, the district implemented a vaccination campaign at all health facilities, conducted extra community outreaches and established mobile vaccination teams. All these activities were implemented under the “Periodic Intensified Immunizations” project, with a focus on children 6–59 months. Sensitization of residents to avoid public gatherings when they develop fever or rash was carried out. By November 2016, the outbreak had been brought under control.

## Additional file

Additional file 1: Case Control Questionnaire: Mayuge District, October 2016. Questionnaire used during the case control investigation in the Mayuge District measles outbreak investigation. (DOCX 36 kb)

## Abbreviations

AR: Attack rate
CDC: Centers for disease control and prevention
CI: Confidence intervals
DHIS2: District health information software 2
EPI Lab: Expanded program on immunization laboratory
HC: Health Centre
IgM: Immunoglobulin M
MoH: Ministry of Health
OR: Odds ratio
OR_MH_: Mantel-Haenszel odds ratio
RR: Relative risk
UVRI: Uganda virus research institute
VC: Vaccination coverage
VE: Vaccine effectiveness
WHO: World Health Organization
