# Supplementary material for: Measles outbreak propagated by children congregating at water collection points in Mayuge District, eastern Uganda, July – October, 2016
Source: BMC Infect Dis. 2018 Aug 20;18:412. doi: 10.1186/s12879-018-3304-5 (PMC6102928; doi:10.1186/s12879-018-3304-5)
Supplement: Supplementary file 1 — Case Control Questionnaire: Mayuge District, October 2016. Questionnaire used during the case control investigation in the Mayuge District measles outbreak investigation. (DOCX 36 kb) [file 12879_2018_3304_MOESM1_ESM.docx]

**Case Control Questionnaire: Mayuge District, October 2016**

**Date of interview…………………………..Name of interviewer …………………………………….. Telephone contact ……………………**

| **Case ascertainment***  **Current or history of** | **Fever**  **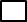Yes 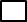 No** | **Generalized rash**  **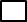Yes 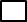No** | **Running nose**  **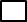Yes 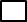No** | | **Cough**  **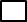Yes 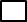No** | **Itching red Eyes**  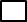**Yes 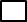No** |
| --- | --- | --- | --- | --- | --- | --- |
| **Respondent status** | - **Case** | | | - **Control** | | |

****case – fever, generalized rash and any of running nose, cough and or itching red eyes***

**Demographic Information**

| **1. Name of patient…………………………………………………….** | **2. Gender: 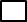 Male 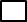 Female** |
| --- | --- |
| **3. DOB…../…/…… or Age completeyrs.……Months…………** | **4. Name of Care taker……………………………………….** |
| **5.Number of people in the household……………………….** | **6. Number of children below 5 years in HH………………….** |
| **7. Village…………………………………………..** | **8. Parish………………………………………………………** |
| **9. Sub-county………………………………………** | **10. District of residence…………………………………** |
| **11. GIS coordinates: Longitude (N)** | **Latitude (E)** |

**CURRENT PRESENTATION (*Read options and tick as applicable*)**

| **Date of onset of Rash (dd/mm/yy)…………………………….…** | | | | | | **Attended Health Facility 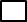Yes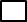No** | | | | | |
| --- | --- | --- | --- | --- | --- | --- | --- | --- | --- | --- | --- |
|  |  |  |  |  |  | **Name of health facility attended** | | | | | |
| **Complications** | **Pneumonia**  **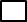Yes 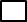No** | | **Ear infection**  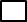**Yes** 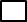**No** | | **Diarrhoea**  **Yes 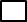 No 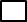** | | **Blindness**  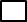**Yes** 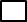**No** | | **Convulsions**  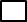**Yes** 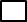**No** | | **Others**  **………………….** |
| **Clinical management**  **and outcome** | | **Vitamin A given**  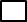**YES** 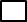**NO** | | **Patient admitted to hosp.**   - **Yes** 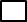**No** | | | | **Recovered**  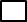**Yes** 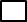**NO** | | **Died**  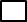**Yes**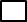**No** | |

| **Nutritional assessment** | **MUAC =** | **TICK THE COLOR:** 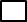**RED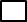GREEN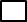 YELLOW** |
| --- | --- | --- |

| **Vaccination history** | **History of any measles vaccination**  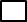**Yes**  **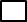 No** | **Received vaccination during**  **Mass campaign** 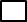**Yes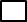 No**  **Routine**  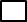**Yes** 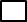**No** | | **Date of last vaccination**  **Date of Routine………/………/…………**  **Mass campaign………/………/……..…** | | **Card available**   - **Yes** - **No** |
| --- | --- | --- | --- | --- | --- | --- |
| **Objection to measles vaccination** | - **YES** 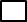**No** | | | **State reasons…………………………………………….**  **………………………………………………………………….** | | |
| **Exposure period** | **Using a calendar month, date of rash onset (Beginning) 21 days………………** | | |  | **Onset (End) 7 days………...** | |
| **Where you in the following places between days stated above** | **School**  **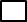Yes** 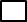 **No** | | **If yes, were there any persons with measles symptoms.** 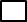**Yes** 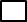 **No** | **If yes, times do you go to school a week ……………..** | **If yes Name place……** | |
|  | **Church**  **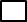Yes 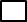No**  **Name:** | | **If yes, was/were there any persons with measles symptoms.**  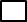**Yes** 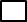**No** | | **No of times that goes to church in a week……….** | |
|  | **Mosque**   - **Yes**   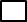**No**  **Where:....** | | **If yes, was/were there any persons with measles symptoms.**  **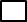Yes 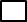No** | | **No of times goes to mosque………………** | |
|  | **Health facility**  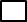 **Yes** 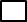**No**  **Name:..** | | **If yes, was/were there any persons with measles symptoms.** 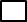**Yes 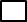No** | | **How many times did you visit the facility?** | |
| **In the same period, were you in any of the following social gatherings** | **Nigina**  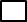**Yes** 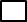**No** | | **If yes, was/were there any persons with measles symptoms.** 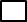**Yes 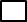No** | | **If yes Name place……** | |
|  | **Saving club**  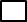**Yes**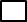 **No**  **Name:** | | **If yes, was/were there any persons with measles symptoms.** 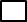**Yes 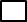No** | | **If yes, No. of times did you go for saving club…..** | |
|  | **Market days**  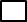**Yes 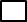No**  **Name:** | | **If yes, was/were there any persons with measles symptoms.** 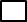**Yes 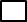No** | | **If yes, No. of times did you go for market days…..** | |
|  | **Burials**  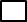**Yes** 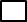**No** | | **If yes, was/were there any persons with measles symptoms.** 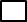**Yes 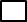 No** | | **If yes Name place……** | |
|  | **Played at the village play ground**  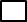**Yes 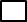No**  **Name:** | | **If yes, was/were there any persons with measles symptoms.**  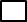**Yes 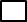 No** | | **IF yes: How many times a week?**  **……………………………….**  **For how long per day**  **……………………………….** | |
|  | **Played in neighbors compound**  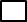**Yes**  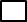 **No**  **IF YES, give name of home……………………………………** | | | **If yes, was/were there any persons with measles symptoms.**  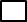**Yes 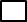 No** | **How many times a week?**  **……………………………**  **How long a day?.................** | |
|  | **Neighbors children played in your compound**  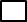**Yes**  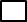**No**  **IF YES, give name of home……………………………………** | | | **If yes, was/were there any persons with measles symptoms.**  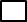**Yes 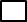 No** | **How many times a week?**  **……………………………**  **How long a day?.................** | |
|  | **Does this child go/collect water either alone or with other children?**  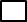 **Yes No** 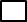  **If yes, where?..........................** | | | **If yes, was/were there any persons with measles symptoms at this water collection point**  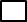 **Yes 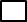 No** | **If yes how many times a day?.................................................** | |
| **Within the above period, have you travelled to the following places?** | **Travel within village**   - **Yes** 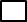**No**   **Name of place?..** | | | **If yes, was/were there any persons with measles symptoms in family you visited? 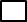Yes 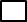No** | | |
|  | **Travel within the parish**   - **Yes** 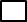**No**   **Name place…** | | | **If yes, was/are there any persons with measles symptoms in place you visited? 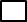 Yes 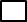 No** | | |
|  | **Travel outside parish**   - **Yes** 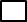**No**   **To where?...** | | | **If yes, was/were there any persons with measles symptoms in place you visited? 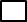 Yes 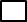 No** | | |
|  | **Travel outside subcounty**  **Yes 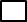 No 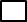**  **To where?..** | | | **If yes, was/were there any persons with measles symptoms in place you visited?** 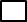**Yes** **No** | | |
|  | **Travel outside district**  **Yes No**  **To where?** | | | **If yes, was/were there any persons with measles symptoms in place you visited? Yes No** | | |

**Laboratory data**

| **Sample** | **Collection date** | **Date received in the lab** | **Type of test** | **Date of result** | **Positive** | **Negative** |
| --- | --- | --- | --- | --- | --- | --- |
|  |  |  |  |  |  |  |

**Final classification**

**Lab confirmed** **Epidemiologically linked** **Clinically classified** **Discarded**
